# Three-Dimensional Printing in Orthopaedic Surgery: A Scoping Review of Technological Innovations, Clinical Applications, Cost-Effectiveness, and Regulatory Challenges (2015-2025)

**DOI:** 10.7759/cureus.110215

**Published:** 2026-06-03

**Authors:** Rabeeia Parwez, Rawaha Husam Al-Deen, Sana Batool, Shahan Shahid, Praveen Rajan

**Affiliations:** 1 Trauma and Orthopaedics, Basildon University Hospital, Basildon, GBR; 2 General Internal Medicine, Quaid-E-Azam Medical College, Bahawalpur, PAK

**Keywords:** 3d printing, additive manufacturing, orthopaedics, patient-specific implants, point-of-care manufacturing, surgical guides

## Abstract

Three-dimensional (3D) printing, or additive manufacturing, has emerged over the past decade as a transformative technology in orthopaedic surgery, offering personalised, anatomically accurate, and biologically integrated solutions across multiple subspecialties. This literature review aimed to map significant technological innovations from 2015 to 2025, consolidate clinical applications, critically appraise cost and feasibility data, and identify regulatory and operational challenges that continue to limit widespread adoption. A comprehensive search of PubMed/MEDLINE, Scopus, Cochrane, Web of Science, and Google Scholar was performed using terms related to 3D printing, additive manufacturing, rapid prototyping, and orthopaedics. Eligible peer-reviewed English-language studies published between January 2015 and 2025 - including randomised controlled trials, cohort and feasibility studies, scoping and systematic reviews, technical reports, and bibliometric analyses - were screened in duplicate, yielding 34 included studies from an initial 11,300 records. A descriptive synthesis was conducted, with data organised across four domains. Major technological advances include the introduction of biodegradable metals (magnesium, calcium, zinc, and iron), metal-matrix composites, highly porous titanium scaffolds promoting osseointegration, and the deployment of point-of-care workflows enabling in-house production of patient-specific implants and bioresorbable devices. Clinical applications span trauma, paediatric orthopaedics, complex arthroplasty, oncology, external fixation, prosthetics, and orthotics, with consistent evidence of reduced operating time, intraoperative blood loss, and fluoroscopy exposure. Reported costs vary widely, from under US$10 to over US$20,000, with economic modelling suggesting net savings when operative-time reductions are accounted for. However, persistent barriers include the absence of harmonised regulatory and quality-control frameworks, high upfront infrastructure and training costs, limited long-term outcome data, and underexplored environmental considerations. Future progress will depend on coordinated efforts between clinicians, engineers, and policymakers to standardise practice, advance multicentre trials, and ensure equitable, sustainable adoption.

## Introduction and background

One of the fast-moving technologies finding use in orthopaedics is three-dimensional (3D) printing. It is a manufacturing technology employed to make a 3D object from a digitally designed model [1]. Also referred to as additive manufacturing, 3D printing is the process of joining materials in a layer-by-layer mechanism to create a final 3D product [2]. Its use is increasing and has been expanding in medical practice over the past 10 years as surgeons and researchers take advantage of the flexibility of the technology to produce objects [3]. In comparison to traditional manufacturing technologies, the primary benefit of 3D printing is the capability to create objects with complex structures and manufacture an assembly in a single-step process [4]. In orthopaedics, it is used in pre-operative planning, surgical simulation, patient-specific instrumentation and implants, bioprinting, prosthetics, and orthotics [5]. It is also resource- and cost-effective, helping with planning and enhancing efficiency in orthopaedic procedures. Moreover, it enhances education and training and offers more affordable prostheses and customised implants for rare cases [6]. 3D printing allows surgeons to better understand patient anatomy, leading to the creation of more elaborate surgical schemes, improved outcomes, increased safety, and improved speed of procedures [7]. The most prevalent applications involve the creation of frameworks and methodologies for diagnosis, training, planned surgery, and the actual production of bone reconstructions and implants.

A bibliometric study was performed to assess the state of research in 3D printing in orthopaedics and traumatology [8]. A total of 412 relevant publications were identified, including 72 publications in 2023, with an annual growth rate of 41.86%. This high growth rate signifies the rising global interest [8]. A second study indicated that most papers were published in China, followed by the United States, the United Kingdom, and India. Considering recent trends, researchers believe that 3D printing is already key to the future of orthopaedics and trauma care [9]. Applications of 3D printing in orthopaedic surgery are increasing rapidly, with trauma and oncology being the most prevalent subspecialties utilising the technology [10]. According to one survey, 53 National Health Service trusts (approximately 25% of those in the UK) are already implementing the technology with a broad range of strategies and applications. Nevertheless, the results showed that there are no specific standards and guidelines related to 3D printing of medical devices, highlighting the importance of clear and consistent regulatory frameworks [11].

Despite the studies reporting on a range of 3D printing technologies, no literature has provided a comprehensive review of significant technical innovations in orthopaedics over the past decade across materials, types of printers, and point-of-care workflows [12]. Second, although clinical applications are well documented, they are not consolidated across subspecialties, with little unified information on how surgeons apply patient-specific models, guides, and implants in daily practice [13]. Third, cost and feasibility information is inconsistent; a scoping review found that reported costs varied from less than US$10 to US$20,000+, with print times ranging from hours to weeks, often without a detailed breakdown of workflow steps [10]. Fourth, quality-control and regulatory issues, including process standardisation and accountability for device safety, remain under-researched, posing a significant obstacle to wider implementation [14]. Finally, there is a lack of high-level evidence on the long-term implementation and sustainability of 3D printing in clinical practice; although early randomised controlled trials are promising, scoping reviews continue to call for further evidence [15].

This review aims to map significant technological changes over the past decade, bring together clinical applications, critically assess cost and feasibility, and identify regulatory and operational challenges, thus bridging an important gap in the understanding of the role of 3D printing in orthopaedics.

## Review

Materials and methods

Study Design

This is a scoping literature review focusing on mapping the technology, clinical uses, and operational issues of 3D printing in orthopaedics over the past ten years. Other factors reviewed include cost and feasibility, and regulatory and quality-control concerns associated with clinical implementation. 

Literature Search Strategy

A comprehensive literature search was conducted across multiple electronic databases, including MEDLINE, Google Scholar, Scopus, Cochrane, and Web of Science, to identify relevant publications. The search strategy combined keywords and Medical Subject Headings (MeSH) terms related to 3D printing, additive manufacturing, rapid prototyping, and orthopaedics. Examples of search terms included "3D printing", "additive manufacturing", "rapid prototyping", "orthopaedics", "trauma", "bones", and "joints". Studies published between 1 January 2015 and 1 January 2025 were included. Boolean operators were applied: ("3D printing" OR "3-dimensional printing" OR "three-dimensional printing" OR "additive manufacturing" OR "rapid prototyping") AND ("orthopaedics" OR "ortho" OR "trauma" OR "bones" OR "joints"). Reference lists of included studies were also screened to identify additional relevant publications. The search was restricted to articles published in English.

Inclusion and Exclusion Criteria

Studies included in this review encompassed the following designs: randomised controlled trials, clinical trials, cohort studies, case-control studies, cross-sectional studies, narrative reviews, systematic reviews, technical reports, and bibliometric analyses. Publications had to be peer-reviewed, written in English, and published within the past 10 years (2015-2025) to reflect the latest technological improvements. Articles investigating the application of 3D printing, additive manufacturing, or rapid prototyping in orthopaedics or trauma-related practice were included. Eligible studies addressed any clinical application, including patient-specific implants, surgical guides, preoperative planning, and oncology models, as well as technical innovation, workflow studies, and cost and feasibility studies.

Studies unrelated to orthopaedics, such as those focused on other specialties (e.g., dentistry, cardiology, neurosurgery, maxillofacial surgery), unless they specifically involved orthopaedic practices, were excluded. Articles published in non-English languages were not considered. Editorials, commentaries, letters, and promotional content lacking substantive data were also excluded.

Study Selection and Data Extraction

The study selection process was conducted in two phases. In stage one, two reviewers (RP and SB) independently screened titles and abstracts to identify potentially relevant studies. In stage two, full-text publications were retrieved and reviewed for eligibility against the inclusion and exclusion criteria. Data were extracted by two reviewers independently (RP and SB), including author, year, country, and study design. Any disagreement was resolved through discussion, and a third review by a more senior author (supervisor of the study: RP) was consulted when necessary.

Data Synthesis

A descriptive synthesis of the included studies was conducted. Mapping of technological advances was performed according to printer types, materials, and point-of-care workflows. Clinical applications were classified by orthopaedic subspecialty. Data on costs and feasibility were summarised, and operational and regulatory issues were critically appraised. Owing to heterogeneity in study designs and outcomes, a meta-analysis was not performed.

Results

Study Selection

**Figure 1:**
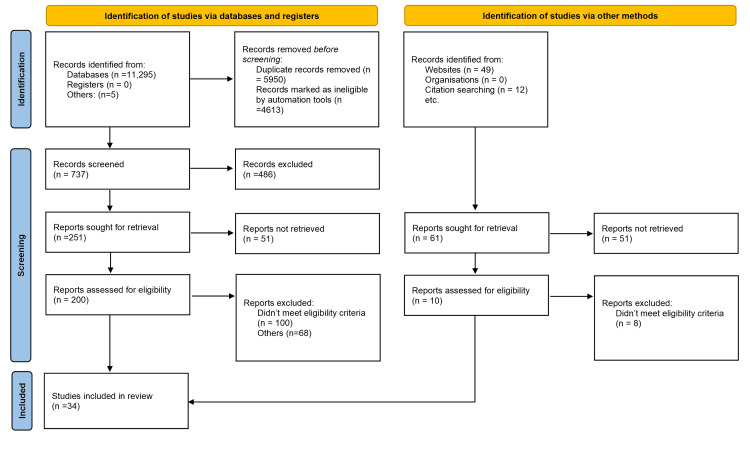
PRISMA flow diagram summarizing the study selection

Database searches and other sources yielded a total of 11,300 records. After 5,950 duplicates and 4,613 records identified through automation tools found on the respective databases were eliminated, 737 records were screened by title and abstract. Data were extracted by two reviewers independently (RP and SB), including author, year, country, and study design. Any disagreement was resolved through discussion, and a third reviewer (RH) was consulted when necessary. Two hundred full-text articles were assessed against the eligibility criteria, and 34 studies were ultimately included in the review (Figure 1).

Major Technological Innovations

In orthopaedics, 3D printing has grown substantially over the past 10 years, with several important technological developments contributing to this trend, including new materials, manufacturing processes, and point-of-care workflows.

Material innovations have been a key focus. Analyses suggest that 70-80% of clinically used implants today are composed of niobium, tantalum, nitinol, titanium alloys, cobalt-chromium alloys, and stainless steels. However, biodegradable metals - including magnesium, calcium, zinc, and iron - have attracted growing attention, with many recent advances reported [16]. Simultaneously, metal matrix composites (MMCs) are under development for orthopaedic implantation owing to their ability to deliver tailored material strength, corrosion resistance, and bioactivity. This has resulted in a new generation of biomaterials with properties unattainable in monolithic counterparts [17]. Point-of-care workflows have proved especially transformative, revolutionising the design of cementless orthopaedic implants through biomimetic, highly porous titanium structures that promote bone ingrowth and osseointegration while reducing stress shielding [18]. It is now possible to produce patient-specific implants within the hospital environment. For example, segmentation software (e.g., Materialise Mimics, Geomagic, nTopology) and on-site printing have been used to fabricate bioresorbable custom plates, meshes, and bone scaffolds [19]. Together, these technological innovations indicate a shift towards more personalised, biologically integrated, and decentralised 3D printing in orthopaedics.

Overview of Clinical Applications

The literature has shown that 3D printing is being incorporated across various orthopaedic subspecialties. Research revealed that pre-operative planning using 3D printing in orthopaedic trauma minimised operating time, intraoperative blood loss, and fluoroscopy time [20]. Another significant application is patient-specific surgical guides. These guides are useful in accurately directing screws, osteotomies, and reductions, enhancing surgical precision and minimising radiation and operative time [21]. Custom 3D-printed joint models support pre-operative planning in both primary and revision arthroplasty. For example, 3D knee models help surgeons simulate implant placement, especially in cases of deformity or bone loss [22]. In paediatric upper- and lower-extremity surgery, 3D printing minimises operating room time, intraoperative blood loss, and radiation exposure [23]. Another use is in external fixation: 3D-printed external fixator frames have been examined in early-stage research on fracture management. Mechanical and clinical case studies indicate that these devices can provide strengths comparable to traditional metal fixators while being tailored to patient morphology [24]. Results also indicated that 3D printing can be used to customise prostheses and orthoses with high accuracy, achieving better anatomical fit, faster recovery, and fewer complications. Shorter production times and lower costs were particularly encouraging in paediatric orthopaedics [25].

Cost and Feasibility Insights

The costs and feasibility of 3D printing in orthopaedics are highly variable and context dependent. A scoping review reported costs from less than US$10 to US$20,000, reflecting variations in printer technology, supplies, workforce, software, and post-processing requirements [10]. Several studies suggest potential cost-effectiveness once clinical benefits are considered. Literature-based financial analyses indicate that medical 3D printing may reduce operating room expenses through reductions in procedure time [26]. The mean estimated time saved per case was 29.9 minutes in the first year of modelling at a hospital, translating to substantial cost savings using a reference operating room cost of US$97 per minute [27]. In practice, one orthopaedic hospital study found that patient-specific anatomical models and guides cost between EUR 150 and EUR 700 per patient when manufactured in-house, depending on complexity [28]. In a narrative review, Parodi et al. emphasised that establishing an in-house 3D printing laboratory in a hospital requires, in addition to printers and post-processing equipment, software licences, a quality-management system, and trained personnel [29]. The unit cost of producing anatomical models can vary widely, from a few dollars for simple structures to hundreds of euros for more complex anatomy, depending on the technology employed [29].

Limitations and Challenges

Although the clinical applications are promising, the literature also exposes several notable challenges that have hampered wider adoption of 3D printing in orthopaedics. First, quality-control and regulatory frameworks remain a significant challenge. Because a large proportion of 3D-printed devices are patient-specific, standard procedures are difficult to implement, and there is no universally accepted standard for validating materials, mechanical strength, biocompatibility, or sterilisation. The absence of unified regulatory systems across jurisdictions further complicates translation into routine practice [30,31]. Another problem is the high initial and operating costs. The lack of formal training programmes for clinicians and engineers is a further limitation, contributing to inconsistent workflows and quality control. The high cost of high-quality printers and biocompatible materials also remains a significant barrier. Scalability and cost are major constraints to feasibility. Rezzadeh et al. noted that the high fixed costs, the requirement for specialised facilities and trained staff, and the need to handle particular materials often lead hospitals to outsource 3D printing to third-party vendors, with consequent lead-time delays [32]. Long-term evidence is also limited; although randomised controlled trials and early studies indicate a positive impact on surgical efficiency, few high-quality studies have measured long-term implant performance, durability, or patient outcomes [15]. Finally, the issue of safety and environmental considerations has not been adequately researched: printer emissions (e.g., volatile organic compounds) and waste-disposal policies remain unregulated, particularly in low- and middle-income settings [32].

Table 1 provides a structured summary of included studies, covering study design, key findings, and critical appraisal.

**Table 1:** Summary of the included studies

Author/Year	Study Design	Key Findings	Strengths/Limitations
O'Connor et al., 2024[1]	Systematic Review & Meta-analysis	Reduced OR time, blood loss, and fluoroscopy exposure; improved surgical accuracy across multiple subspecialties	Strong high-level evidence; inter-study variability limits generalisability
Wixted et al., 2021 [2]	Narrative Review	Overview of current 3D printing applications and future directions in orthopaedic surgery	Broad clinical scope; lacks quantitative data and statistical synthesis
Wong, 2016[3]	Narrative Review	Foundational overview of patient-specific 3D-printing applications and clinical utility in orthopaedics	Seminal early reference; evidence base predates recent technological innovations
Lee et al., 2016[4]	Technical Study	Demonstrates core additive manufacturing principles; referenced for definitional context in orthopaedic literature	Innovative manufacturing insights; limited direct clinical relevance; included for foundational context
Papagelopoulos et al., 2018 [5]	Narrative Review	Comprehensive overview of 3D technologies in orthopaedics, including implants, surgical guides, and anatomical models	Wide scope and well-structured; primarily descriptive with no quantitative analysis
Hasan et al., 2019[6]	Narrative Review	Highlights cost-effective 3D printing applications in orthopaedic surgery for low-resource settings	Valuable low-resource context; findings may not transfer to well-resourced healthcare systems
Frizziero et al., 2019[7]	Clinical Study	3D printing in paediatric orthopaedic surgery improves surgical outcomes and reduces OR time and costs	Clinically relevant paediatric outcomes; small cohort size
Rojas et al., 2025[8]	Bibliometric Study	Rapid global growth: 412 publications identified; annual growth rate of 41.86%; China and the USA leading output	Large bibliometric dataset; no direct clinical correlation or outcome measurement
Vaishya et al., 2018 [9]	Bibliometric Study	Analyses publication trends and knowledge mapping in 3D printing across orthopaedic literature	Useful overview of research landscape; partially outdated given rapid field growth
Levesque et al., 2020 [10]	Scoping Review	Costs range from under US$10 to over US$20,000; wide variation in print times; highlights reporting inconsistency	Broad scope covering multiple applications; inconsistent cost reporting limits direct comparison
Ul Azeem et al., 2024 [11]	Observational Study	53 NHS trusts (~25% of UK) using 3D printing; wide strategy variation; no unified regulatory standards identified	Real-world national adoption data; limited external validity beyond the UK NHS context
Beitler et al., 2022 [12]	Narrative Review	Identifies regulatory factors governing point-of-care 3D printing in hospital and medical centre settings	High policy relevance; primarily guidance-focused with limited primary clinical data
Teo et al., 2021 [13]	Feasibility Study	Demonstrates the feasibility of point-of-care 3D printing workflows for orthopaedic trauma in a hospital setting	Practical real-world workflow insights; limited by small scale and single-centre design
Akpa-Inyang et al., 2025 [15]	Scoping Review	Identifies ethical, legal, and regulatory challenges in the application of 3D printing in orthopaedics	Comprehensive ethical and regulatory scope; highlights the ongoing lack of standardization
Wong et al., 2021[15]	Scoping Review	Limited high-quality RCT evidence available; calls for long-term studies on implant durability and patient outcomes	Important evidence-gap identification; few high-quality RCTs exist to draw firm conclusions
Liang et al., 2023[16]	Narrative Review	Reviews advances in 3D printing of biodegradable metals (Mg, Ca, Zn, Fe) for orthopaedic applications	Strong focus on emerging biomaterials; limited translation to clinical practice
Khalid et al., 2025[17]	Technical Study	Assesses additively manufactured metal-matrix composites (MMCs) as next-generation orthopaedic implant materials	Innovative materials approach; early-stage evidence with limited long-term clinical data
Tigani et al., 2025 [18]	Clinical Study	Highly porous 3D-printed titanium implants promote osseointegration in cementless off-the-shelf joint replacement	Clinically relevant innovation; limited follow-up period and small patient cohort
Maintz et al., 2024 [19]	Technical Study	Point-of-care production of patient-specific bioresorbable polymer implants; details material, technology, and scope	Demonstrates real-world point-of-care capability; limited scale and early clinical experience
Morgan et al., 2020 [20]	Systematic Review & Meta-analysis	Preoperative 3D printing reduces OR time, intraoperative blood loss, and fluoroscopy in orthopaedic trauma surgery	High-level evidence for trauma applications; inter-study variability in reporting
Hess et al., 2024 [21]	Systematic Review & Meta-analysis	3D-printed surgical guides demonstrate high accuracy for screws, osteotomies, and reductions across subspecialties	Strong methodology and broad application scope; inter-study protocol variability
Rosso et al., 2022 [22]	Clinical Study	3D-printed preoperative models improve planning accuracy in complex primary and revision total knee arthroplasty	Directly clinically applicable; limited by small cohort size
Mounsef et al., 2024[23]	Systematic Review	3D printing-guided preoperative planning improves outcomes in paediatric upper and lower extremity procedures	Focused and clinically relevant paediatric review; inter-study variability among included studies
O'Connor HA et al., 2023 [24]	Systematic Review	3D-printed external fixator frames demonstrate mechanical performance comparable to traditional metal fixators	Good synthesis of emerging technology; limited number of available clinical trials
Pelczarski et al., 2025 [26]	Narrative Review	3D printing enables customised prostheses and orthoses with improved anatomical fit, comfort, and reduced recovery time	Clinically useful overview; lacks quantitative outcome data and direct comparisons
Ballard et al., 2019 [26]	Economic Analysis	Financial modelling shows 3D printing reduces OR costs through decreased procedure time in orthopaedic surgery	Economically relevant methodology; dependent on modelling assumptions and reference cost rates
Ravi et al., 2023[27]	Economic Analysis	Average of 29.9 minutes OR time saved per case in year one of hospital 3D printing; significant cost savings identified	Real-world academic hospital data; single-centre design limits external validity
Kveller et al., 2024 [28]	Observational Study	In-house 3D printing costs EUR150–EUR700 per patient model; documents workflow from a first-year hospital facility	Practical first-hand cost data; limited generalisability beyond single-centre Scandinavian setting
Parodi et al., 2025 [29]	Narrative Review	Guides in-house hospital 3D printing lab setup, covering equipment, software, staffing, and unit cost considerations	Practical clinical guidance; no standardized cost benchmarks provided
Neussl et al., 2025 [30]	Systematic Review	Reviews quality approaches and validation standards for 3D printing in orthopaedic and traumatological settings	Rigorous quality-focused analysis; limited consensus across institutions and jurisdictions
Cong et al., 2025[31]	Narrative Review	Reviews innovative 3D printing technologies and advanced biomaterials and their applications in orthopaedic surgery	Forward-looking and comprehensive; novel materials remain early-stage in clinical validation
Rezzadeh et al., 2020 [32]	Economic Analysis	Economic analysis highlights high fixed costs and outsourcing implications for patient-specific 3D printing in hospitals	Clinically grounded economic insight; relies on theoretical modelling with variable assumptions
Awuah et al., 2023[33]	Perspective	Explores 3D printing potential for trauma and fracture care in LMICs; highlights access barriers and resource constraints	Highlights underserved global context; perspective-based with limited empirical data

Discussion

This review synthesises 10 years of advances in 3D printing in orthopaedics, including technological progress, clinical applications, cost and feasibility considerations, and the limitations that continue to constrain its use. Over the past decade, 3D printing has moved from experimental research into routine clinical application, opening new possibilities for personalised, biologically integrated, and patient-specific solutions.

The development of biomaterials has been one of the most radical changes in orthopaedics. Conventional implants use alloys of titanium, cobalt-chromium, nitinol, and stainless steel. Recent advances in biodegradable metals - such as magnesium, calcium, and zinc - offer temporary structural support that gradually resorbs, potentially eliminating the need for implant removal [16]. At the same time, MMCs and porous titanium scaffolds have allowed the production of implants with improved osseointegration, regulated stiffness, and mechanical bone-matching, addressing long-standing problems such as stress shielding and implant loosening [17,18]. Orthopaedic model and implant design have been radically transformed by point-of-care workflows. Segmentation software, such as Materialise Mimics, Geomagic, and nTopology, now enables hospitals to create patient-specific models, guides, and even bioresorbable scaffolds [19]. The rapid manufacture of these devices supports improved pre-operative planning, surgical rehearsal, and precision-guided operations, especially in trauma, paediatric orthopaedics, and complex joint replacement [22,23].

Another key finding of this review is that 3D printing has been widely adopted in clinical practice. Pre-operative models enhance operative efficiency, reducing operating time, intraoperative blood loss, and fluoroscopy exposure [20]. Patient-specific surgical guides for osteotomies, spinal instrumentation, and screw placement increase precision and reproducibility while reducing the influence of human factors [21]. Paediatric applications particularly benefit from lower radiation doses and improved fixation. In addition, 3D-printed orthoses and prostheses offer superior anatomical fit, increased comfort, and shorter recovery times compared with conventional devices [23]. Early work on 3D-printed external fixator frames demonstrates the potential to mechanically reproduce existing metal-based frames while allowing tailoring to patient anatomy [24].

Cost and feasibility remain important considerations. Prices for orthopaedic models and implants vary widely, from US$10 or less for a basic planning model to more than US$20,000 for advanced implants requiring high-end printers and biocompatible materials [10]. Economic studies suggest that the technology may prove cost-efficient through reductions in operating room time and costs; one study reported a mean of 29.9 minutes saved per case, translating to a substantial reduction in operating room expenditure [26,27]. Nevertheless, initial investment, staff training, and workflow integration remain significant barriers, particularly in low-resource settings. Outsourcing 3D printing to third parties may also create lead-time delays, making same-day point-of-care production less realistic [29,30].

Despite these developments, several limitations and challenges remain. Regulatory and quality-control systems for patient-specific 3D-printed devices are still underdeveloped, with no established standards for validating mechanical strength, biocompatibility, or sterilisation [30,31]. Variability in segmentation methods, printer calibration, and operator skill can affect reproducibility, and specialised training programmes for clinicians and engineers remain limited [30,32]. There is also a paucity of long-term evidence on implant durability, clinical outcomes, and cost-effectiveness [15]. Environmental and occupational implications, such as printer emissions and the disposal of polymer waste, are under-reported, raising safety and sustainability concerns in the hospital environment [33].

Several converging directions are evident for the future of orthopaedic 3D printing. First, there is a need for harmonised regulations and uniform quality-control standards to guarantee safety and reproducibility. Second, next-generation biomaterials - including functionally graded and "smart" implants - should be developed to enhance mechanical performance and biological integration. Third, automation in segmentation, design, and post-processing will reduce labour costs, expand throughput, and broaden access. Fourth, robust multicentre clinical trials are needed to provide long-term insights into implant performance, patient outcomes, and cost-efficiency. Finally, affordability and sustainability must be improved, particularly for low- and middle-income countries, through low-cost printers, recycled materials, and regional print hubs.

Orthopaedic 3D printing is at a critical juncture: technological advances have made personalised, biologically inspired care possible; clinical interventions have demonstrated clear practical benefits in trauma, paediatric, and reconstructive settings; and point-of-care workflows have become more efficient. However, high costs, regulatory gaps, insufficient long-term evidence, and sustainability concerns must be resolved before routine, large-scale adoption is achievable. The transformative potential of 3D printing in orthopaedic care will be best realised through coordinated efforts across the clinical, engineering, and regulatory domains.

## Conclusions

Over the past decade, 3D printing has emerged as a transformative technology in orthopaedic surgery, enabling personalised, anatomically accurate, and biologically integrated solutions. Technological advances in biomaterials, metal-matrix composites, biodegradable metals, and point-of-care workflows have supported the routine production of patient-specific implants, surgical guides, anatomical models, and customised prostheses, with consistent clinical benefits demonstrated in pre-operative planning, surgical accuracy, and reductions in operating time, intraoperative bleeding, and radiation exposure - particularly in paediatric and complex cases.

However, routine large-scale adoption remains constrained by high initial costs, variable institutional feasibility, the absence of harmonised regulatory and quality-control frameworks, limited long-term outcome data, and underexplored sustainability considerations. Realising the full potential of 3D printing in orthopaedics will require coordinated efforts between clinicians, engineers, and policymakers to standardise quality and regulatory standards, advance multicentre clinical trials, and develop affordable, scalable point-of-care solutions that ultimately translate technological promise into improved patient outcomes.
